# The Good, the Bad, and the Ugly: The Influence of Skull Reconstructions and Intraspecific Variability in Studies of Cranial Morphometrics in Theropods and Basal Saurischians

**DOI:** 10.1371/journal.pone.0072007

**Published:** 2013-08-08

**Authors:** Christian Foth, Oliver W. M. Rauhut

**Affiliations:** 1 SNSB, Bayerische Staatssammlung für Paläontologie und Geologie, Richard-Wagner-Str. 10, Munich, Germany; 2 Department of Earth and Environmental Sciences, Ludwig-Maximilians-University, Richard-Wagner-str. 10, Munich, Germany; 3 GeoBioCenter, Ludwig-Maximilians-University; Richard-Wagner-str. 10, Munich, Germany; Raymond M. Alf Museum of Paleontology, United States of America

## Abstract

Several studies investigating macroevolutionary skull shape variation in fossil reptiles were published recently, often using skull reconstructions taken from the scientific literature. However, this approach could be potentially problematic, because skull reconstructions might differ notably due to incompleteness and/or deformation of the material. Furthermore, the influence of intraspecific variation has usually not been explored in these studies. Both points could influence the results of morphometric analyses by affecting the relative position of species to each other within the morphospace. The aim of the current study is to investigate the variation in morphometric data between skull reconstructions based on the same specimen, and to compare the results to shape variation occurring in skull reconstructions based on different specimens of the same species (intraspecific variation) and skulls of closely related species (intraspecific variation). Based on the current results, shape variation of different skull reconstructions based on the same specimen seems to have generally little influence on the results of a geometric morphometric analysis, although it cannot be excluded that some erroneous reconstructions of poorly preserved specimens might cause problems occasionally. In contrast, for different specimens of the same species the variation is generally higher than between different reconstructions based on the same specimen. For closely related species, at least with similar ecological preferences in respect to the dietary spectrum, the degree of interspecific variation can overlap with that of intraspecific variation, most probably due to similar biomechanical constraints.

## Introduction

Recent years have seen an increase in studies on macroevolutionary patterns of skull shape in fossil reptiles using geometric morphometrics (e.g. [1–6]). However, undistorted, complete, and three-dimensionally preserved skulls are an exception in fossil taxa. Thus, in all of these studies the sampling of skulls was based mainly on reconstructed skulls and at least partly on reconstructions taken from the scientific literature. However, this approach could be potentially problematic as a) skull reconstructions might differ considerably due to incompleteness and/or deformation of the material, and b) the influence of intraspecific variation is partly ignored in these macroevolutionary approaches, as is ontogenetic variation in most cases (with the exception of the study of Bhullar et al. [3]). The quality of the reconstructions is crucial, because the position of landmarks on reconstructed skulls as well as the position of species within the morphospace depends on the shape of the whole cranium and the precise relations between its individual bones. Furthermore, the position of species within the morphospace may also vary due to intraspecific variation. In the past some studies have tried to quantify intraspecific variation in dinosaur skulls with the help of morphometric and geometric morphometric methods, e.g. [7–12], whereas variation caused by taphonomic deformation was well-documented by Carpenter [7] and Chapman [9]. However, a comprehensive review of the variability of morphometric data due to differential reconstructions or as a result of intraspecific variation for any dinosaur lineage has not been published yet.

The aim of the current study is to investigate the variation in morphometric data between skull reconstructions based on the same specimen with the help of geometric morphometric methods. We furthermore analysed which skull regions might particularly be affected by high variation within these reconstructions. The results are compared to shape variation occurring in skull reconstructions based on different specimens of the same species and skulls of closely related species, in order to investigate whether this potential source of variation in geometric morphometric data might be comparable to taxonomically or even phylogenetically significant variation.

## Material and Methods

Three different datasets for basal Saurischia, basal Tetanurae, and Tyrannosauroidea were created, by collecting skull reconstructions in lateral view (Table S1 in File S1). The taxon sample was, of course, limited to taxa for which several skulls are known and for which various reconstructions based on the same specimen could be found in the literature. All datasets include a) skull reconstructions based on the same specimen, b) skull reconstructions of different specimens of the same species and c) skull reconstruction of closely related species. *Plateosaurus* and *Allosaurus* were treated as each being represented by a single species, following Weishampel & Chapman [13], Möser [14] and Carpenter [8]. The specimen FMNH PR308, which was originally described as *Gorgosaurus* [15], is placed in *Daspletosaurus* , following Carr [16].

**Figure 1:**
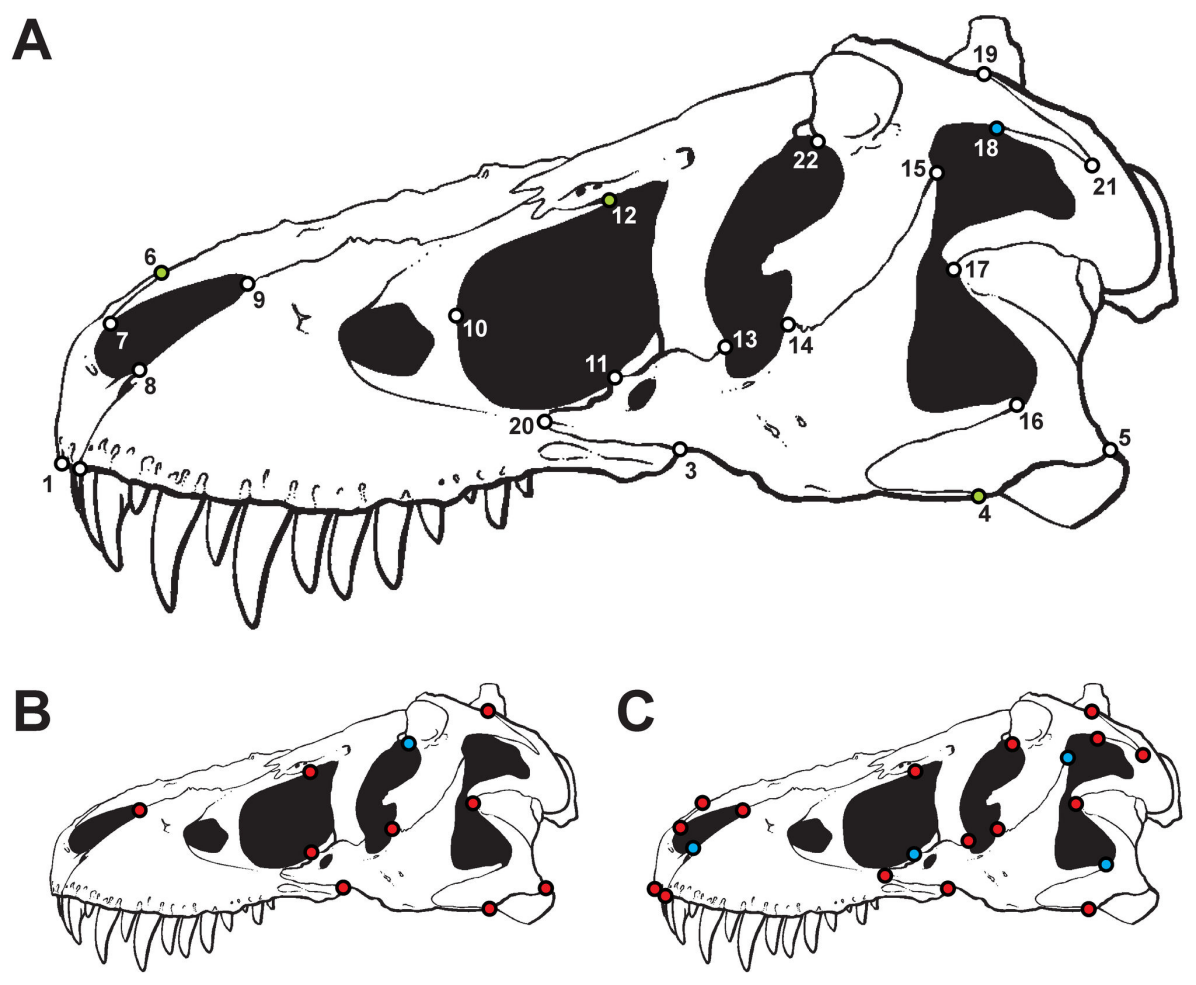
Position of landmarks used in the study and variation of skull regions. A: Landmarks used in the study plotted on a skull reconstruction of *Tyrannosaurus* specimen of AMNH 5027 (modified after Carr & Williamson [60]). The green landmarks show skull regions that show most variation between different reconstructions based on the same specimen in both the original and the randomized dataset. The blue landmark LM 18 shows additional variation found in the original dataset. B: Skull regions with distinct variation between reconstructions based on different specimens (intraspecific variation). Red landmarks show variation found in both the original and the randomized dataset, blue landmarks show variation found in the randomized dataset. C: Skull regions with distinct variation between reconstructions based on different, closely related species (interspecific variation). Red landmarks show variation found in both the original and the randomized dataset, blue landmarks show variation found in the randomized dataset.

The skull shape of all species/specimens was captured by 22 homologous landmarks, which are figured in Figure 1 and listed in Table S2 in File S1, using the program tpsDig [17]. This program outputs a tps (thin plate spline) file with two-dimensional landmark coordinates and scale (size) data for each specimen. The tps file was loaded into MorphoJ [18] and superimposed using Generalized Procrustes Analyses GPA, which align landmarks from all specimens by minimizing non-shape variation like size, location, orientation and rotation [19].

Afterwards, the datasets were divided into different subgroups containing the Procrustes coordinates of a) single specimens, b) different specimens of the same species and c) different, closely related species, respectively. To estimate the degree of variation of skull shape within single specimens, species and between different species a method was used that was originally developed for estimating the methodological error for plotting landmarks on specimens by hand [20]. On the basis of the Procrustes coordinates the mean Procrustes distances to the respective consensus coordinates of each landmark were calculated. Then, the relation of these distances to the mean distance of the consensus landmarks to the centroid of the consensus shape was calculated as a percentage of the former from the latter. A further tps file was created for each dataset including a single skull reconstruction of only one specimen (n = 10) to calculate the methodological error of plotting landmarks on the skull reconstruction as mentioned above. The mean error for plotting landmarks (= 0.364%) was computed and subtracted from the percentage errors for individual landmarks. Afterwards, the median of the percentage error of each landmark and its 25^th^ and 75^th^ percentiles (interquartile range) were computed in PAST 2.17b [21] and compared between the different subgroups. Using this method for the purpose mentioned above, the results do not represent methodological errors, but a measure for morphological variation of overall shape (disparity, see 22,23). If the median is more than 5.0% skull shape, variation within a sample was considered as significant. Thus, skull reconstructions from sample with significant variation could potentially affect the results of a geometric morphometric analyses and should be treated with caution. To verify the results, Procrustes coordinates were additionally used to calculate the Euclidian distances for every sample within each group [24]. As in the previous case, the median Euclidian distance and its 25^th^ and 75^th^ percentiles were calculated.

Furthermore, we wanted to know, which skull regions are particularly affected by significant shape variation within reconstructions of the same specimen, the same species and closely related taxa, respectively. For this, the median and its 25^th^ and 75^th^ percentiles were calculated for each landmark within the different subgroups mentioned above.

Due to the generally small numbers of skull reconstructions for most samples, we tested the robustness of the ‘original’ results in relation to sample size by computing random samples in the program R [25] with a standard number of ten ‘hypothetical reconstructions’ per sample on the basis of the Procrustes coordinates of the original data. The function used computed ten normal pseudorandom variates based on the mean and the standard deviation of all Procrustes coordinates related to a corresponding landmark within the original sample [26]. Afterwards, all methods described above were repeated with randomized samples and compared to the original data. If both kinds of data produce similar results one can conclude that the results of the original data are robust in relation to sample size.

## Results

**Figure 2:**
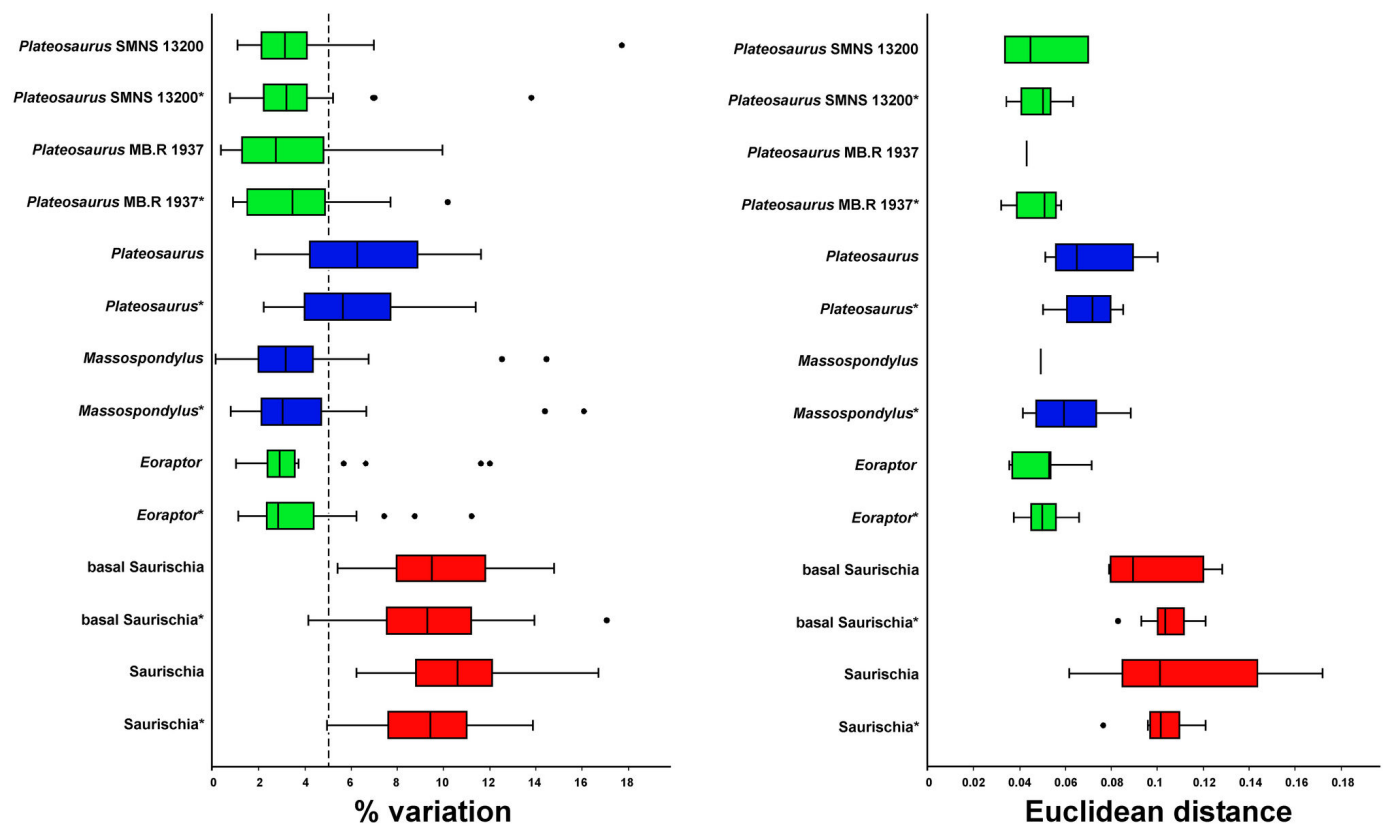
Percentage variation and Euclidean distance for different skull reconstructions and randomized skull shapes of basal Saurischia. Shaded boxes show the interquartile range (defined by the 25^th^ and 75^th^ percentile) with the median marked as horizontal line. The whiskers mark the distance between the interquartile range and points up to 1.5 distances from the interquartile range. Outliers are represented as circles. Green boxes show shape variation between reconstructions based on the same specimen, blue boxes show shape variation between reconstructions based on different specimens (intraspecific variation), and red boxes show shape variation between reconstructions based on different, closely related species (interspecific variation). (*) Randomized samples.

**Figure 3:**
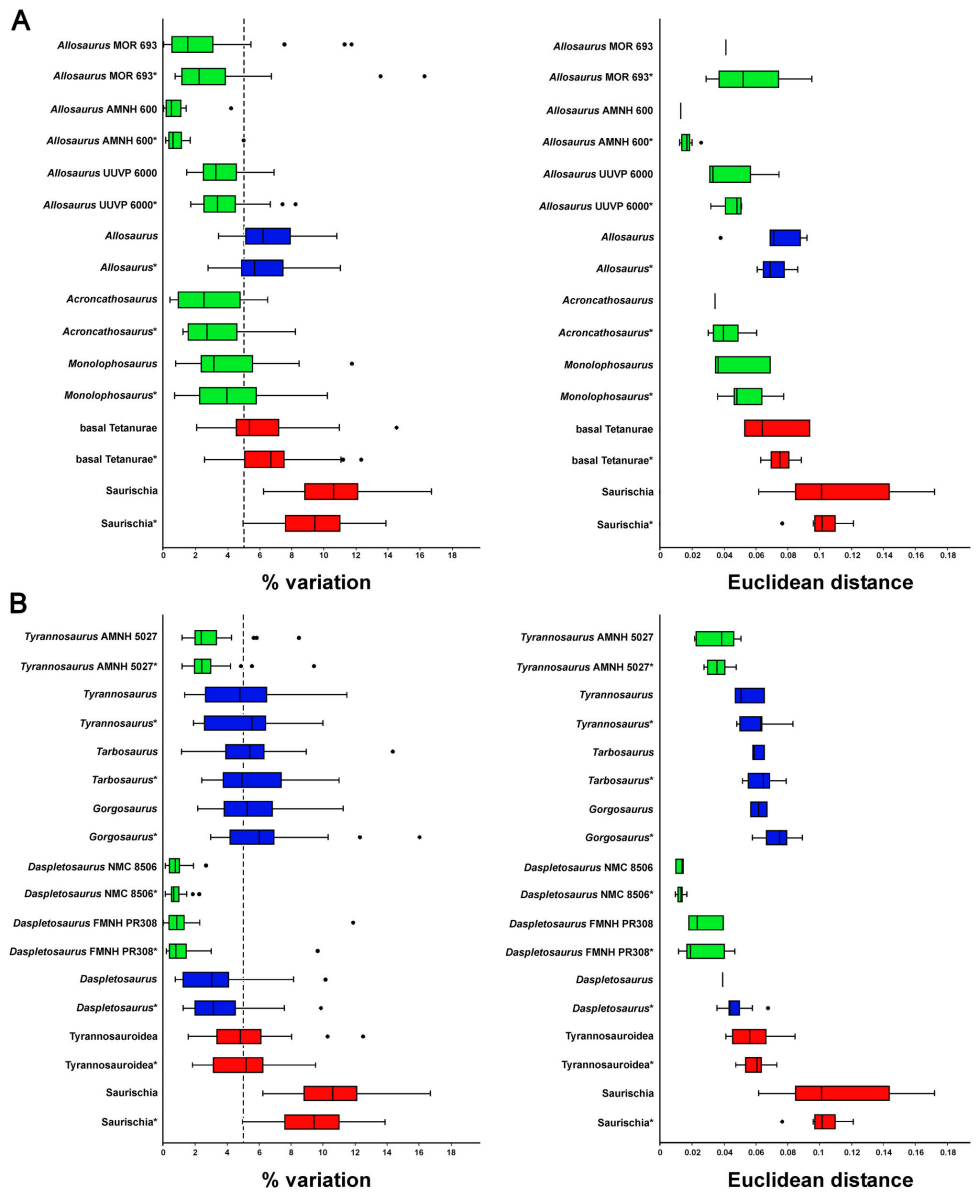
Percentage variation and Euclidean distance for different skull reconstructions and randomized skull shapes of basal Tetanurae and Tyrannosauroidea. A: basal Tetanurae. B: Tyrannosauroidea. Shaded boxes show the interquartile range (defined by the 25^th^ and 75^th^ percentile) with the median marked as horizontal line. The whiskers mark the distance between the interquartile range and points up to 1.5 distances from the interquartile range. Outliers are represented as circles. Green boxes show shape variation between reconstructions based on the same specimen, blue boxes show shape variation between reconstructions based on different specimens (intraspecific variation), and red boxes show shape variation between reconstructions based on different, closely related species (interspecific variation). (*) Randomized samples.

Both the values of the median of landmark variation (median of variation) and Euclidean distances show generally similar distributions between the single samples of the three subgroups. This is also true for the comparison between original and randomized data. However, for the Euclidean distances the interquartile range of the randomized data is usually smaller than for the original data (for all samples with more than two reconstructions) with exception of *Gorgosaurus* , *Tarbosaurus* and the *Daspletosaurus* specimen FMNH PR308. In contrast, the range of interquartiles are comparable for both kinds of data with the exception of *Eoraptor* *,* *Massospondylus* and *Tarbosaurus* (here the interquartile range of the randomized data is slightly bigger than in the original data) as well as *Plateosaurus* , *Acrocanthosaurus* and the *Tyrannosaurus* specimen AMNH 5027 (here the interquartile range of the randomized data is slightly smaller than in the original data, Figures 2, 3).

In all sampled cases the median of the variation for reconstructions based on the same specimen is less than 5.0%. In the *Allosaurus* specimen AMNH 600 (two reconstructions) and the *Daspletosaurus* specimens NMC 8506 (four reconstructions) and FMNH PR308 (three reconstructions) the median of variation is even less than 1.0%. The mean for the median values of the original data is 2.08% (and 2.27% for the randomized data). Only in *Monolophosaurus* is the 75^th^ percentile value higher than 5.0% both original and randomized data.

The mean of the median values for skull reconstructions based on different specimens of the same species is 4.74% for the original data (and 4.78% for the randomized data), in which the median of the variation of the original data is less than 5.0% for *Daspletosaurus* , *Massospondylus* and *Tyrannosaurus* . Here, the 75^th^ percentile value is less than 5.0% in the former two genera as well. Thus, the median of the variation of *Daspletosaurus* and *Massospondylus* strongly overlaps with that of reconstructions based on the same specimen for most taxa. In contrast, the median of the variation of the original data of *Allosaurus* , *Plateosaurus* , *Tarbosaurus* and *Gorgosaurus* is more than five percent, but only for *Allosaurus* is the 25^th^ percentile value higher than five percent. In contrast, the median of the variation in the randomized datasets is less than 5.0% for *Tarbosaurus* , but more than 5.0% in *Allosaurus* , *Plateosaurus* *,* *Gorgosaurus* *and* *Tyrannosaurus* (Figures 2, 3).

The mean of the median values for reconstructions of skulls of closely related taxa is 6.48% for the original data (and 6.76% for the randomized data). For the original data of Tyrannosauroidea only the 75^th^ percentile value is more than five percent, whereas median of the randomized data is more than 5.0% as well. For basal Tetanurae the median of variation is more than 5.0%. Thus, the degree of variation (in relation to the interquartile range) of both basal Tetanurae and Tyrannosauroidea overlaps with that of reconstructions based on skulls of the same species. Only for basal Saurischia and all Saurischia sampled are the medians of the variation and their percentiles considerably higher than 5.0%. In the latter cases the median of the variation is over 9.0%, and thus, distinctly higher than that for basal Tetanurae and Tyrannosauroidea (Figures 2, 3). All results shown in Figures 2 and 3 are summarized in Table S3, Table S4 and Table S5 in File S1.

For reconstructions based on the same specimen most variation can be seen in the ventral contact of the jugal and quadratojugal (LM 4), the contact between premaxilla and nasal along the dorsal margin of skull (LM 6), the position of the most anterior point of the lacrimal along the dorsal margin of the antorbital fenestra (LM 12), and the contact between postorbital and *squamosum* along the dorsal margin of the lateral temporal fenestra (LM 18, but only for the original data), as the 75^th^ percentile of values the percentage variation is more than 5.0% for these landmarks (Figure 1, Table S6, Table S7 in File S1).

For reconstructions based on different specimens of the same species distinct variation occurs in the ventral margin of the jugal and its contacts with the maxilla and quadratojugal (LM 3, LM 4), the position of the posteroventral corner of the quadratojugal (LM 5), the length of tip of the maxillary process of the nasal (LM 9), in the position of the most ventral point of the lacrimal along the margin of the antorbital fenestra (LM 11), the position of the anteriormost contact of the lacrimal along the dorsal margin of the antorbital fenestra (LM 12), the contact between lacrimal and jugal on the orbital margin (LM 14), the position of the anteroventral tip of the ventral process of the squamosal on the margin of the lateral temporal fenestra (LM 17), and in the dorsal contact between postorbital and squamosal (LM 19). For the randomized data the contact between frontal and postorbital on the dorsal margin of the orbit (LM 22) was found to be significant as well (Figure 1, Table S6, Table S7 in File S1).

In comparison, for skull reconstructions of closely related taxa, distinct landmark variation affects almost the entire skull, with the exception of the length of the anterior process of the maxillary body (LM 8), the position of the anteriormost point of the antorbital fenestra (LM 10), the contact of the jugal with both the squamosal and the quadratojugal on the margin of the lateral temporal fenestra (LM 15, LM 16). For the randomized data all landmarks except of LM 10 showed significant variation (Figure 1, Table S6, Table S7 in File S1).

## Discussion

**Figure 4:**
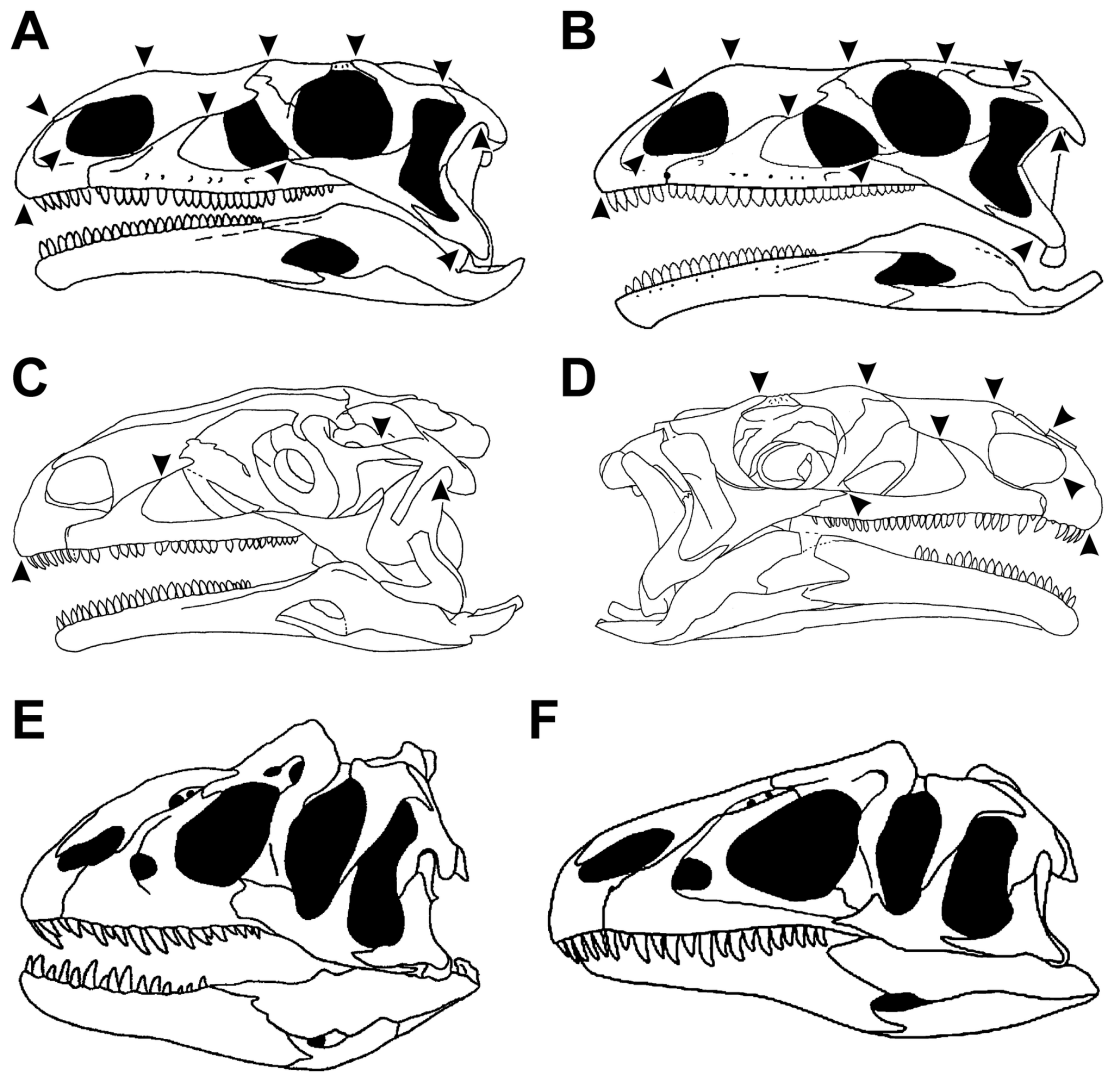
Skull reconstructions of the *Plateosaurus* specimen SMNS 13200 and different *Allosaurus* specimens. A: Skull reconstruction of SMNS 13200 after Galton [31]. B: Skull reconstruction of the SMNS 13200 after Yates [30] (modified after Nesbitt [51]). C: Line drawing of the left side of the original material of SMNS 13200 after Galton [61]. D: Line drawing of the right side of the original material of SMNS 13200 after Galton [61]. Arrows show shape differences in the reconstructions by Galton and Yates and the morphology of the respective structure of the original material of SMNS 13200. Here, the skull reconstruction of SMNS 13200 by Galton resembles the original material more in respect to the shape of the anterior margin of the premaxilla and its contact to the nasal, the shape of the anterior margin of the external naris, the contact between nasal and maxilla, the contact between maxilla, jugal and lacrimal, the shape of the dorsal margin of the skull, the shape of the postorbital and its contacts to the frontal and the *squamosum*, and the shape of the ventral margin of the quadratojugal. E: ‘Short-snouted’ *Allosaurus* specimen USNM 4735 described by Gilmore [61], which was based on a disarticulated, partially deformed, and pathological skull (modified after Henderson [62]). F: ‘Typical’ *Allosaurus* skull based on MOR 693 (modified after Rauhut [29]).

Based on the results presented above, we can conclude that the shape variation of skull reconstruction (in relation to the median of variation and the interquartile range) based on the same specimen seems usually to be negligible in geometric morphometric studies (only in *Monolophosaurus* the 75^th^ percentile is more than 5.0%). The general consistency of the results between original and randomized data supports this result in spite of the small sample sizes of the original data. However, taxa for which only a single specimen and maybe even only a single reconstruction exist could introduce considerable error in geometric morphometric studies, if the particular specimen is incomplete or strongly taphonomically deformed. In *Allosaurus* , for example, the skull reconstructed by Gilmore [27] has figured prominently in both the scientific and the popular literature for a long time, until newly found, better preserved and complete specimens showed that this reconstruction, based on a disarticulated, partially deformed, and pathological skull, does not represent the “typical” skull shape of this taxon (Figure 4).

Shape variation in reconstructions might be influenced, for instance, by the talent of the artists, their anatomical knowledge and their tendency to idealize structures, which are e.g., taphonomically deformed, damaged or missing (meaning to attempt a complete de-deformation of the skull). Differences in the skull shape of the holotype of *Monolophosaurus* or the *Plateosaurus* specimens MB.R 1937 and SNMS 13200 are probably partially caused by the latter factor, because Zhao & Currie [28], Rauhut [29] and Yates [30] idealized such deformations more completely than Galton [31] (Figure 4) or Brusatte et al. [32] (e.g. Brusatte et al. figured the disarticulation between jugal and postorbital on the right side of the skull). Furthermore, it might be important if the artist saw the specimen first hand, reconstructed the skull on the basis of photographs or simply redrew the skull from previously published reconstructions (as is the case e.g. with the reconstruction of *Monolophosaurus* in Rauhut [29]). In order to minimize this source of error, a scientist analysing shape changes would be wise to not only take the reconstructed skull from the literature, but also look closely at the available data on the original material and how the skull was reconstructed from it.

Within different reconstructions based on the same specimens the skull regions described by landmark 4, 6, 12 and 18 (i.e. the ventral contact of the jugal and quadratojugal, the contact premaxilla and nasal along the dorsal margin of skull, the position of the most anterior point of the lacrimal along the dorsal margin of the antorbital fenestra, and the contact between postorbital and *squamosum* along the dorsal margin of the lateral temporal fenestra) are more variable than other landmarks, although their variability is still less than that between landmarks in reconstructions of different specimens. Thus, these particular skull regions may contain a potential methodological error for plotting landmarks on dinosaur skulls and maybe also other reptiles, and should be verified carefully by photo material or first-hand observations.

The variation of skull reconstructions (in relation to the mean of the median values) based on different specimens of the same species is expected to be higher than that of different reconstructions of the same specimen as variation is further caused by intraspecific variation. However, the differences are not significant due to the strong overlap of the percentiles between both groups and also vary from species to species. For instance, the intraspecific skull variation found in *Massospondylus* is relatively low, challenging Gow et al. [33], who hypothesized that the shape variation seen in the skulls of two *Massospondylus* specimens might be caused by sexual dimorphism. Based on the results of both the original and randomized samples this hypothesis cannot be supported statistically. The variation might rather reflect allometric shape variation as both specimens slightly differ in skull size [34]. Cranial sexual dimorphism was also hypothesized for *Allosaurus* [35], but also cannot be verified statistically.

On the other hand, the current results support previous studies on *Allosaurus* and *Plateosaurus* , which show a large intraspecific variation within these taxa [8,13,36]. However, some of the variation presented in those studies reflects also ontogenetic variation, making a direct comparison of the studies difficult as this type of variation has only minor impact on the current results due to selective sampling of adult or nearly adult specimens.

Some of the variation found in the current results may also result from taphonomic deformation (e.g. the disarticulated contact of quadratojugal and *squamosum* in the holotype skull PIN 551-1 of *Tarbosaurus* , which is pictured in the reconstruction of Maleev [37]). Taphonomic deformation was also hypothesized as the major reason for the huge ‘morphological variation’ seen in the southern Germany *Plateosaurus* material [14], and its influence on skull shape is well-documented for a *Plateosaurus* by Chapman [9]. Furthermore, some variation in *Allosaurus* and *Plateosaurus* could be also explained by their controversial taxonomic status. As mentioned in the material and method section, reconstructed skulls of both genera were treated as belonging to one species, but some authors argued that there are at least two species for each genus (e.g. [30,31,38–40]). If the latter case is true, the variation is partially covered by interspecific variation, and thus the actual intraspecific variation might be overestimated.

To minimize the ‘error’ of intraspecific variation in macroevolutionary approaches, taxa for which there are several good quality reconstructions of different specimens should be tested for intraspecific variation. This can be done in a separate small dataset with the same landmark configuration used in the macroevolutionary study by calculating the Procrustes coordinates for each specimen and estimating the respective Euclidean distances to the consensus shape of the small dataset. Subsequently, the specimen with the smallest distance to the consensus shape might be used for the study.

The examples of interspecific variation (in relation to the median of variation) presented in this study show all significant variation, except for the original sample of Tyrannosauroidea. However, the latter exception could be the result of a small sample size (n = 5). Interestingly, the interspecific shape variation (in relation to the interquartile range) of basal Tetanurae and Tyrannosauroidea strongly overlaps with the shape variation of the intraspecific variation of *Allosaurus* , *Tyrannosaurus* , *Tarbosaurus* and *Gorgosaurus* . The estimated intraspecific variation is even slightly higher than the estimated interspecific variation of the respective groups. At first glance this result is surprising, as one would expect that interspecific variation should be larger than intraspecific variation, as seen in basal Saurischia. Methodically, the overlap could be a false signal resulting from small sample sizes (see 41). However, the differences between the numbers of reconstructions used for a single species and for different, closely related species are rather small, making this explanation rather unlikely. Furthermore, because the results of the randomized data are similar to that of the original one, the sample size does not seem to influence the current result significantly. However, it is possible that the chosen landmark configuration does not capture skull regions that underlie strong interspecific variation in basal Tetanurae or Tyrannosauroidea, like the dorsal margin of the nasal (e.g. *Monolophosaurus* ) or the dorsal margin of the lacrimal horn (e.g. *Allosaurus* ). Furthermore, semi-landmark analysis of overall skull shape, in combination with a landmark-based analysis, might capture variations in skull shape more completely and thus yield different results. Thus, it is possible that the present analyses underestimate the actual interspecific variation between those taxa. Furthermore, it is to be expected that interspecific skull variability increases with increasing the sample size of taxa analysed, as it is indeed demonstrated by the higher variation seen in the data set for basal saurischians or saurischians as a whole. By expanding the data set to species with more derived skull morphologies (e.g. long-snouted spinosaurids for basal Tetanurae), an increase of the interspecific variation even in rather closely related forms would also be expected. This is supported by several studies on crustacean, pterosaur and coelurosaur diversity for instance, which all show that disparity of larger taxonomic clades is higher than in the respective internal subclades (see 4,42–44). On the other hand, an overlap of intraspecific and interspecific variation in closely related taxa has also been demonstrated for instance in the cranial shape of recent Hominoidea [24], the osteology of skinks [45] or in molecular sequences of different bilaterian clades (e.g. [46–48]), and the phenomenon is therefore neither restricted to theropod dinosaurs, nor to skull shape.

In comparison with this rather small variation seen in closely related theropod taxa, basal Saurischia in total possess a very large interspecific variation. One reason for this could be the inclusion of *Eoraptor* , the taxonomic position of which is still debated, e.g. [49–51]. However, excluding *Eoraptor* from the data set does not change the result (median of variation = 9.66%). Thus, the large variation seen in the skull shape might be due to diverging dietary preferences in basal saurischians, towards carnivory in many basal theropods, with omnivory and finally herbivory in sauropodmorphs [52–55]. Indeed, this change in diet might lead to the evolutionary trend from slender and elongate skulls to short and broad skulls seen in the early evolution of Sauropodomorpha [56]. A similar pattern regarding diet preferences was also found in theropods by Brusatte et al. [2] and Foth & Rauhut [6], who have shown that both carnivorous and non-carnivorous taxa occupy large areas within the morphospace, but non-carnivorous taxa tend to develop more diverse, sometimes aberrant skull morphologies (e.g. Oviraptorosauria). In contrast, large-bodied carnivorous theropods tend to cluster closely together within morphospace [2,6], and show a smaller disparity in skull shape in comparison to smaller theropods with a broad dietary spectrum [2]. This might be due to a constrained biomechanical adaptation for high bite forces [57–59], including an oval orbit, a deep jugal body and a short postorbital region [6,58].

## Conclusion

The median of variation of different skull reconstructions based on the same specimen seems to have generally little influence on the results of a geometric morphometric analysis of skull shape in theropods and basal saurischians. Shape differences seem to be mainly influenced by the talent of the artists, their anatomical knowledge, and their tendency to idealize structures that are damaged, missing or taphonomically deformed. In general, it is advisable to verify reconstructions used on the basis of the original material or photographs thereof. For different specimens of the same species the variation (in relation to the mean of the median values) is generally higher than in the previous example, indicating that intraspecific variation cannot be neglected, although this apparent variation might in some cases be overestimated due to uncertain taxonomy. For closely related species, at least with similar ecological preferences, the degree of interspecific variation (in relation to the median of variation and its percentiles) overlaps with that of intraspecific variation. This probably reflects considerable constraints in the skulls of theropods with similar feeding strategies. As would be expected, variation in morphometric data might increase with increased phylogenetic and/or ecological sampling, but this have to be tested in future studies in more detail. Given the nature of fossil data, our analysis is necessarily based on rather small sample sizes, and more investigations of the relation between intraspecific and interspecific variation in geometric morphometric data in recent animals, for which higher sample sizes are available, would be desirable.

## Supporting Information

File S1 **Including institutional abbreviations, sources of skull reconstructions, *Allosaurus* specimens, description of landmarks and error of landmarks**
(PDF)

File S2 **Including geometric morphometric data for MorphoJ (txt).** Data also deposited at Dryad: http://datadryad.org. Accessed 2013 July 15. doi:10.5061/dryad.6ss84.
(TXT)
