# Supplementary material for: The Good, the Bad, and the Ugly: The Influence of Skull Reconstructions and Intraspecific Variability in Studies of Cranial Morphometrics in Theropods and Basal Saurischians
Source: PLoS One. 2013 Aug 8;8(8):e72007. doi: 10.1371/journal.pone.0072007 (PMC3738521; doi:10.1371/journal.pone.0072007)
Supplement: File S1 — Including institutional abbreviations, sources of skull reconstructions, Allosaurus specimens, description of landmarks and error of landmarks (PDF) [file pone.0072007.s001.pdf]

Supporting information S1

The good, the bad, and the ugly: the influence of skull reconstructions and intraspecific variability in studies of cranial morphometrics in theropods and basal saurischians.

by Christian Foth, Oliver W.M. Rauhut

Institutional abbreviations

AMNH, American Museum of Natural History, New York; BHI, Black Hills Institute, Hill City; BP, Bernard Price Institute for Palaeontological Research, University of the Witwatersrand, Johannesburg; FMNH, The Field Museum, Chicago; GPIT, Geologisch-Paläontologisches Institut, Tübingen (IFGT Institut für Geowissenschaften, Eberhard-Karls-Universität, Tübingen); IVPP, Institute of Vertebrate Palaeontology and Palaeoanthropology, Beijing; LACM, Los Angeles County Museum, Los Angeles; MB, Museum für Naturkunde, Berlin; MOR, Museum of the Rockies, Bozeman; NM, National Museum, Bloemfontein; NMC, National Museum of Canada, Ottawa; NMMNH, New Mexico Museum of Natural History and Science, Albuquerque; NCSM, North Carolina Museum of Natural Sciences, Raleigh; PIN, Paleontological Institute, Russian Academy of Sciences, Moscow; PVSJ, Museo de Ciencias Naturales, Universidad Nacional de San Juan, San Juan; QMNS, Qatar Museum of Nature and Science; SMA, Sauriermuseum, Aathal; SMNS, Staatliches Museum für Naturkunde Stuttgart, Stuttgart; TMP, Royal Tyrrell Museum of Palaeontology, Drumheller; TTU, Texas Tech University, Lubbock; ULBRA, Museu de Ciências Naturais, Universidade Luterana do Brasil, Canoas; USNM, National Museum of Natural History (= formerly United States National Museum),

Smithsonian Institution, Washington, D.C.; UUVP, Utah Museum of Natural History, Salt Lake City; ZPAL, Institute of Palaeobiology, Polish Academy of Sciences, Warsaw.

Table S1. List of specimens used in the study

Taxon	Collection number	Reference
**Allosauroidea + basal Tetanurae**		
Acrocanthosaurus atokensis	NCSM 14345	Currie & Carpenter 2000; Eddy & Clarke 2011
Allosaurus spp.	TTU P9269	McClelland 1990
	AMNH 600	Osborn 1903; Molnar et al. 1990
	DINO 11541	Chure 2000
	MOR 693	Rauhut 2003; Foth & Rauhut, this study
	UUVP 6000	Madsen 1976; Molnar et al. 1990; Holtz et al. 2004; Paul 2002; 2008; Fastovsky & Weishampel 2005; Westheide & Rieger 2009; Foth & Rauhut, this study
	SMA 0005	Foth & Rauhut, this study
	QMNS-FO-456	Foth & Rauhut, this study
Monolophosaurus jiangi	IVPP 84019	Zhao & Currie 1993; Rauhut 2003; Brusatte et al. 2010
Sinoraptor dongi	IVPP 10600	Currie & Zhao 1993
**Sauropodomorpha + basal Saurischia**		
Eoraptor lunensis	PVSJ 512	Sereno et al. 1993; Rauhut 2003; Langer 2004; Paul 2002; Martinez et al. 2011, Nesbitt 2011
Massospondylus carinatus	BP/l/4934	Gow et al. 1990
	BP/l/5241	Gow et al. 1990
Melanorosaurus	NM QR3314	Yates 2007
Pampadromaeus barberenai	ULBRA-PVT016	Cabreira et al. 2011
Plateosaurus engelhardti	AMNH 6810	Galton 2001
	Composite	Wilson & Sereno 1998;
	GPIT 1	Galton 2001
	MB R. 1937	Galton 2001; Rauhut 2003
	SMNS 12949	Galton 2001
	SMNS 13200	Galton 2001, Yates 2003; Nesbitt 2011

**Taxon**	**Collection number**	**Reference**
**Tyrannosauroidea**		
Bistahieversor sealeyi	NMMNH P-27469	Carr & Williamson 2010
Daspletosaurus torosus	NMC 8506	Russell 1970; Molnar 1990; Holtz 2004, Paul 2008
	FMNH PR308	Russell 1970; Molnar et al. 1990; Rauhut 2003
Gorgosaurus libratus	composite	Carr 1999; Paul 2008
	TMP 91.36.500	Carr et al. 2011
Tarbosaurus bataar	PIN 551-1	Maleev 1974; Paul 2008
	ZPAL MgD−I/4	Hurum & Sabath 2003
Tyrannosaurus rex	AMNH 5027	Osborn 1912; Carpenter 1992; Molnar et al. 1990; Carr & Williamson 2004; Holtz 2004; Paul 2008; 2010
	BHI 3033	Larson 1997
	LACM 23844	Carr & Williamson 2004

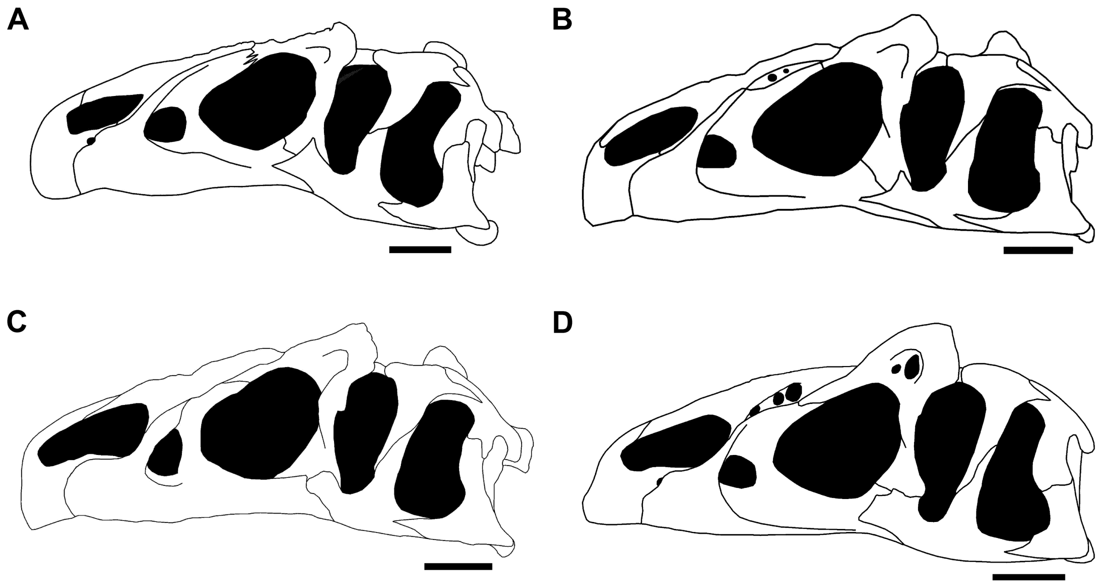

Figure S1. Additional skull reconstructions of some *Allosaurus* specimens used for this study. A: UUVP 6000. B: MOR 693. C: SMA 0005. D: QMNS-FO-456. Scale bar = 10 cm.

Table S2. Description of landmarks used for the different dataset.

No	Landmark
1	anteroventral corner of the premaxilla
2	contact of premaxilla and maxilla along the tooth row
3	contact of maxilla and jugal along the ventral margin of the skull
4	contact between jugal and quadratojugal along the ventral margin of the skull
5	posteroventral corner of the quadratojugal
6	contact of premaxilla and nasal along the dorsal margin of the skull
7	contact of premaxilla and nasal along the dorsal margin of the external naris
8	tip of the maxillary process of the premaxilla
9	tip of the maxillary process of the nasal
10	most-anterior point of the antorbital fenestra
11	ventralmost point of the lacrimal along the margin of the antorbital fenestra
12	anteriormost contact of the lacrimal along the dorsal margin of the antorbital fenestra
13	contact between lacrimal and jugal on the orbital margin
14	contact between postorbital and jugal on the orbital margin
15	contact between postorbital and jugal on the margin of the lateral temporal fenestra
16	contact between jugal and quadratojugal on the margin of the lateral temporal fenestra
17	anteroventral tip of the ventral process of the squamosal on the margin of the lateral temporal fenestra
18	ventral contact of postorbital and squamosal on the margin of the lateral temporal fenestra
19	dorsal contact between postorbital and squamosal
20	anteriormost point of the jugal
21	posteriormost point of the postorbital
22	contact between frontal and postorbital on the dorsal margin of the orbit

Table S3. Variation of different skull reconstructions within basal Saurischia.

	Percentage error			Euclidean distance		
	**Median**	**25 prcntil**	**75 prcntil**	**Median**	**25 prcntil**	**75 prcntil**
**Plateosaurus SMNS 13200**	2.965	2.114	4.152	0.045	0.034	0.070
**Plateosaurus SMNS 13200***	3.143	2.163	4.332	0.049	0.040	0.055
**Plateosaurus MB.R 1937**	2.605	1.220	4.863	0.043	NA	NA
**Plateosaurus MB.R 1937***	3.062	1.495	5.337	0.051	0.039	0.056
**Plateosaurus**	5.978	4.179	8.981	0.063	0.055	0.092
**Plateosaurus***	5.466	3.906	7.733	0.071	0.060	0.081
**Massospondylus**	3.148	1.919	4.369	0.049	NA	NA
**Massospondylus***	3.042	2.052	4.920	0.058	0.046	0.074
**Eoraptor**	2.910	2.363	3.600	0.052	0.037	0.058
**Eoraptor***	2.806	2.363	4.382	0.048	0.044	0.056
**basal Saurischia**	9.471	7.730	11.894	0.090	0.079	0.124
**basal Saurischia***	9.181	7.347	11.401	0.103	0.098	0.112
**Saurischia**	10.437	8.793	12.167	0.098	0.084	0.146
**Saurischia***	9.288	7.567	11.136	0.101	0.097	0.110

NA = not available due to small sample size, (*) Randomized dataset.

Table S4. Variation of different skull reconstructions within basal Tetanurae

	**Percentage error**			**Euclidean distance**		
	**Median**	**25 prcntil**	**75 prcntil**	**Median**	**25 prcntil**	**75 prcntil**
**Allosaurus MOR 693**	1.466	0.483	3.289	0.041	NA	NA
**Allosaurus MOR 693***	2.122	1.091	4.076	0.048	0.036	0.078
**Allosaurus AMNH 600**	0.437	0.161	1.074	0.013	NA	NA
**Allosaurus AMNH 600***	0.481	0.306	1.218	0.016	0.013	0.019
**Allosaurus UUVP 6000**	3.271	2.444	4.615	0.034	0.031	0.053
**Allosaurus UUVP 6000***	3.321	2.479	4.507	0.048	0.041	0.051
**Allosaurus**	6.209	5.131	8.014	0.071	0.069	0.088
**Allosaurus***	5.636	4.842	7.467	0.067	0.064	0.078
**Acroncathosaurus**	2.309	0.894	4.818	0.034	NA	NA
**Acroncathosaurus***	2.595	1.526	4.609	0.039	0.032	0.049
**Monolophosaurus**	3.057	2.298	5.710	0.036	0.035	0.069
**Monolophosaurus***	3.649	2.246	5.916	0.048	0.045	0.066
**basal Tetanurae**	5.316	4.507	7.301	0.064	0.056	0.086
**basal Tetanurae***	6.289	5.040	7.778	0.074	0.069	0.082
**Saurischia**	10.437	8.793	12.167	0.098	0.084	0.146
**Saurischia***	9.288	7.567	11.136	0.101	0.097	0.110

NA = not available due to small sample size, (*) Randomized dataset.

Table S5. Variation of different skull reconstructions within Tyrannosauroidea

	**Percentage error**			**Euclidean distance**		
	**Median**	**25 prcntil**	**75 prcntil**	**Median**	**25 prcntil**	**75 prcntil**
**Tyrannosaurus AMNH 5027**	2.284	1.969	3.396	0.039	0.023	0.046
**Tyrannosaurus AMNH 5027***	2.410	1.902	3.088	0.035	0.029	0.041
**Tyrannosaurus**	4.794	2.396	6.468	0.051	0.047	0.065
**Tyrannosaurus***	5.483	2.566	6.449	0.062	0.050	0.066
**Tarbosaurus**	5.310	3.924	6.454	0.059	0.058	0.065
**Tarbosaurus***	4.904	3.659	7.474	0.063	0.055	0.071
**Gorgosaurus**	5.140	3.721	7.119	0.062	0.057	0.067
**Gorgosaurus***	5.813	4.131	7.111	0.073	0.066	0.081
**Daspletosaurus NMC 8506**	0.680	0.399	1.047	0.014	0.011	0.014
**Daspletosaurus NMC 8506***	0.649	0.501	1.006	0.012	0.011	0.014
**Daspletosaurus FMNH PR308**	0.842	0.328	1.312	0.023	0.018	0.039
**Daspletosaurus FMNH PR308***	0.745	0.378	1.456	0.018	0.016	0.041
**Daspletosaurus**	2.614	1.197	4.133	0.039	NA	NA
**Daspletosaurus***	3.100	1.876	4.721	0.044	0.043	0.052
**Tyrannosauridae**	4.656	3.269	6.191	0.056	0.043	0.076
**Tyrannosauridae***	4.805	3.049	6.232	0.060	0.053	0.064
**Saurischia**	10.437	8.793	12.167	0.098	0.084	0.146
**Saurischia***	9.288	7.567	11.136	0.101	0.097	0.110

NA = not available due to small sample size, (*) Randomized dataset.

Table S6. Errors of single landmarks with same specimens, same species and different species based on original data

	Same specimen			Same species			Different species		
**Lm**	**Median**	**25 prcntil**	**75 prcntil**	**Median**	**25 prcntil**	**75 prcntil**	**Median**	**25 prcntil**	**75 prcntil**
**1**	1.993	0.715	4.232	4.553	1.442	6.018	10.964	1.570	12.597
**2**	1.900	0.314	2.199	3.551	2.655	6.203	5.362	2.988	10.998
**3**	2.798	0.742	4.842	5.481	3.403	9.436	8.029	4.702	8.487
**4**	3.069	1.908	8.477	8.766	6.434	14.366	10.278	8.147	14.768
**5**	1.828	1.016	3.280	6.136	4.617	6.808	6.365	5.989	9.655
**6**	4.774	2.191	5.677	1.797	1.375	9.554	13.762	4.820	14.532
**7**	1.732	1.395	3.767	3.927	2.363	5.902	5.052	2.305	10.105
**8**	1.283	0.417	2.632	3.930	2.384	7.241	3.963	2.005	10.713
**9**	1.664	0.425	2.904	8.770	3.186	10.795	8.571	5.361	13.805
**10**	1.595	0.829	2.695	3.418	1.138	3.942	3.594	3.365	11.800
**11**	1.263	0.914	2.751	5.142	4.396	5.411	4.793	2.408	9.496
**12**	3.147	0.583	7.000	6.477	4.092	7.909	5.851	4.240	9.447
**13**	2.610	1.169	3.349	4.062	1.475	4.543	5.976	5.271	6.349
**14**	2.423	0.785	3.998	6.302	3.675	8.460	6.007	5.361	6.415
**15**	1.448	0.737	2.347	2.933	1.618	3.412	4.543	4.050	8.494
**16**	2.389	1.419	3.325	4.780	3.458	6.217	4.547	3.362	5.421
**17**	2.064	0.501	4.113	6.262	3.881	6.912	7.705	4.491	7.971
**18**	3.342	0.385	5.682	4.808	2.008	6.202	6.912	2.052	7.008
**19**	2.388	2.147	4.096	5.419	4.422	10.006	9.027	4.399	12.499
**20**	1.490	0.500	3.127	3.947	1.553	6.280	6.116	4.040	6.227
**21**	2.476	0.893	3.041	4.176	2.411	5.221	7.167	4.202	8.405
**22**	1.436	0.593	3.560	4.830	2.282	8.926	5.522	5.419	12.177

Table S7. Errors of single landmarks with same specimens, same species and different species based on randomized data

	Same specimen			Same species			Different species		
**LM**	**Median**	**25 prcntil**	**75 prcntil**	**Median**	**25 prcntil**	**75 prcntil**	**Median**	**25 prcntil**	**75 prcntil**
**1**	1.666	0.824	4.409	4.365	2.496	5.749	10.085	1.878	12.322
**2**	1.685	0.387	2.779	3.465	2.536	4.641	5.691	1.828	13.685
**3**	3.466	0.622	4.764	7.360	3.681	9.149	9.077	4.955	9.267
**4**	3.585	1.864	7.711	9.497	7.826	10.585	11.065	6.266	17.061
**5**	2.657	0.922	3.950	5.421	4.888	5.594	7.388	5.988	9.407
**6**	4.208	2.280	7.424	2.576	1.802	8.819	11.198	2.800	12.019
**7**	1.957	1.498	3.577	4.228	2.546	4.557	6.699	2.101	10.596
**8**	1.545	0.717	2.340	3.959	2.869	6.588	5.845	2.398	13.942
**9**	1.735	1.108	3.168	7.070	3.309	9.997	7.024	3.794	9.284
**10**	1.644	1.365	2.793	3.601	1.901	5.867	4.081	3.434	7.593
**11**	1.447	0.956	3.624	5.631	4.649	5.954	5.068	3.132	8.056
**12**	3.718	0.750	6.943	7.686	6.490	8.352	7.091	5.180	12.322
**13**	2.212	1.280	4.137	3.123	2.830	5.328	6.819	6.704	7.243
**14**	2.421	1.384	3.261	5.614	4.113	7.530	6.220	4.141	8.572
**15**	1.972	1.089	2.886	2.966	2.774	3.990	5.452	5.214	8.439
**16**	2.303	1.768	3.569	4.054	3.810	6.236	5.587	4.329	5.654
**17**	1.936	0.895	4.506	5.747	4.361	7.446	5.235	4.234	9.283
**18**	2.511	1.446	4.374	4.741	3.047	5.830	5.767	2.559	7.523
**19**	2.711	1.662	4.776	6.402	4.786	7.614	9.518	5.879	9.652
**20**	1.617	0.817	3.945	3.821	1.971	4.423	6.446	4.091	8.773
**21**	2.785	0.787	3.440	4.921	3.439	5.300	7.513	4.429	8.633
**22**	2.337	0.703	3.460	6.012	2.188	8.738	6.104	3.587	11.195

References

1. Brusatte SL, Benson RBJ, Currie PJ, Zhao X (2010) The skull of *Monolophosaurus jiangi* (Dinosauria: Theropoda) and its implications for early theropod phylogeny and evolution. Zoological Journal of the Linnean Society 158: 573–607.
2. Cabreira SF, Schultz CL, Bittencourt JS, Soares MB, Fortier DC, et al. (2011) New stem-sauropodomorph (Dinosauria, Saurischia) from the Triassic of Brazil. Naturwissenschaften 98: 1035–1040.
3. Carpenter K (1992) Tyrannosaurids (Dinosauria) of Asia and North America. In: Mateer NJ, Chen P, editors. Aspects of nonmarine Cretaceous geology. Beijing: China Ocean Press. pp. 250–268.
4. Carr TD (1999) Craniofacial ontogeny in Tyrannosauridae (Dinosauria, Coelurosauria). Journal of Vertebrate Paleontology 19: 497–520.
5. Carr TD, Williamson TE (2004) Diversity of late Maastrichtian Tyrannosauridae (Dinosauria: Theropoda) from western North America. Zoological Journal of the Linnean Society 142: 479–523.
6. Carr TD, Williamson TE (2010) *Bistahieversor sealeyi*, gen. et sp. nov., a new tyrannosauroid from New Mexico and the origin of deep snouts in Tyrannosauroidea. Journal of Vertebrate Paleontology 30: 1–16.
7. Carr TD, Williamson TE, Britt BB, Stadtman K (2011) Evidence for high taxonomic and morphologic tyrannosauroid diversity in the Late Cretaceous (Late Campanian) of the American Southwest and a new short-skulled tyrannosaurid from the Kaiparowits formation of Utah. Naturwissenschaften 98: 241–246.

8. Chure DJ (2000) A new species of *Allosaurus* from the Morrison Formation of Dinosaur National Monument (UT-CO) and a revision of the theropod family Allosauridae. Department of Earth and Environmental Sciences, Columbia University. pp.1-909.

9. Currie PJ, Zhao X (1993) A new carnosaur (Dinosauria, Theropoda) from the Jurassic of Xinjiang, People's Republic of China. Canadian Journal of Earth Sciences 30: 2037–2081.

10. Currie PJ, Carpenter K (2000) A new specimen of *Acrocanthosaurus atokensis* (Theropoda, Dinosauria) from the Lower Cretaceous Antlers Formation (Lower Cretaceous, Aptian) of Oklahoma, USA. Geodiversitas 22: 207–246.

11. Eddy DR, Clarke JA (2011) New information on the cranial anatomy of *Acrocanthosaurus atokensis* and its implications for the phylogeny of Allosauroidea (Dinosauria: Theropoda). PLoS ONE 6: e17932.

12. Fastovsky DE, Weishampel DB (2005) The evolution and extinction of the dinosaurs. Cambridge, New York, Melbourne, Madrid, Cape Town, Singapore, Sâo Paulo: Cambridge University Press.

13. Galton PM (2001) The prosauropod dinosaur *Plateosaurus* Meyer, 1837 (Saurischia: Sauropodomorpha; Upper Triassic). II. Notes on the referred species. Revue de Paléobiologie 20: 435–502.

14. Gow CE, Kitching JW, Raath MA (1990) Skulls of the prosauropod dinosaur *Massospondylus carinatus* Owen in the collections of the Bernand Price Institute for Palaeontological Research. Palaeontologia Africana 27: 45–58.

15. Holtz TRJ (2004) Tyrannosauroidea. In: Weishampel DB, Dodson P, Osmólska H, editors. The Dinosauria. Berkeley: University of California Press. pp. 111–136.

16. Holtz TRJ, Molnar RE, Currie PJ (2004) Basal Tetanurae. In: Weishampel DB, Dodson P, Osmólska H, editors. The Dinosauria. Berkeley: University of California Press. pp. 71–110.

17. Hurum JH, Sabath K (2003) Giant theropod dinosaurs from Asia and North America: skulls of *Tarbosaurus bataar* and *Tyrannosaurus rex* compared. Acta Palaeontologica Polonica 48: 161–190.

18. Langer MC (2004) Basal Saurischia. In: Weishampel DB, Dodson P, Osmólska H, editors. The Dinosauria. Berkeley: University of California Press. pp. 25–46.

19. Larson PL (1997) The king's new clothes: a fresh look at *Tyrannosaurus rex*. In: Wolberg DL, Stumps E, Rosenberg GD, editors. Dinofest International. Philadelphia: Academy of Natural Sciences. pp. 65–71.

20. Madsen JH (1976) *Allosaurus fragilis*: a revised osteology. Utah Geological and Mineralogical Survey Bulletin 109: 3–163.

21. Maleev EA (1974) Giant carnosaurs of the family Tyrannosauridae. Trudy Sovmestnaya Sovetsko-Mongolskaya Paleontologicheskaya Ekspeditsiya 1: 132–191.

22. Martinez RN, Sereno PC, Alcober OA, Colombi CE, Renne PR, et al. (2011) A basal dinosaur from the dawn of the dinosaur era in southwestern Pangaea. Science 331: 206–210.

23. McClelland BK (1990) Anatomy and kinesis of the *Allosaurus* skull. Department of Geosciences, Texas Tech University. Pp1-123.

24. Molnar RE, Kurzanov SM, Dong Z (1990) Carnosauria. In: Weishampel DB, Dodson P, Osmólska H, editors. The Dinosauria. Berkeley: University of California Press. pp. 169–209.

25. Nesbitt SJ (2011) The early evolution of archosaurs: relationships and the origin of major clades. Bulletin of the American Museum of Natural History 352: 1–292.

26. Osborn HF (1903) The skull of *Creosaurus*. Bulletin of the American Museum of Natural History 19: 697–701.

27. Osborn HF (1912) Crania of *Tyrannosaurus* and *Allosaurus*. Memoirs of the American Museum of Natural History 1: 1–30.

28. Paul GS (2002) Dinosaurs of the air: the evolution and loss of flight in dinosaurs and birds. Baltimore: John Hopkins University Press.

29. Paul GS (2008) The extreme lifestyle and habits of the gigantic tyrannosaurid superpredators of the Late Cretaceous of North America and Asia. In: Larson P, Carpenter K, editors. *Tyrannosaurus rex*, the tyrant king. Bloomington: Indiana University Press. pp. 307–352.

30. Paul GS (2010) The Princeton field guide to dinosaurs. New Jersey: Princeton University Press.

31. Rauhut OWM (2003) The interrelationships and evolution of basal theropod dinosaurs. Special Papers in Palaeontology 69: 1–213.

32. Russell DA (1970) Tyrannosaurs from the Late Cretaceous of western Canada. National Museum of Natural Sciences, Publications in Palaeontology 1: 1–34.

33. Sereno PC, Forster CA, Rogers RR, Monetta AM (1993) Primitive dinosaur skeleton from Argentina and the early evolution of Dinosauria. Nature 361: 64–66.

34. Westheide W, Rieger R (2004) Spezielle Zoologie. Teil 2: Wirbel- oder Schädeltiere. Heidelberg: Spektrum Akademischer Verlag.

35. Wilson JA, Sereno PC (1998) Early evolution and higher-level phylogeny of sauropod dinosaurs. Society of Vertebrate Paleontology Memoir 5: 1–68.

36. Yates A (2003) The species taxonomy of the sauropodomorph dinosaurs from the Löwenstein Formation (Norian, Late Triassic) of Germany. Palaeontology 46: 317–337.

37. Yates A (2007) The first complete skull of the Triassic dinosaur *Melanorosaurus* Haughton (Sauropodomorpha: Anchisauria). Special Papers in Palaeontology 77: 9–55.

38. Zhao X, Currie PJ (1993) A large crested theropod from the Jurassic of Xinjiang, People's Republic of China. Canadian Journal of Earth Sciences 30: 2027–2036.
